# Weight changes in Portuguese patients with depression: which factors are involved?

**DOI:** 10.1186/1475-2891-13-117

**Published:** 2014-12-16

**Authors:** Jerónima Correia, Paula Ravasco

**Affiliations:** Laboratório de Nutrição da Faculdade de Medicina da Universidade de Lisboa e Hospital Universitário de Santa Maria, Avenida Prof. Egas Moniz, 1649-028 Lisbon, Portugal; Psychiatric Department of the Hospital da Nossa Senhora do Rosário – EPE, Barreiro, Portugal

**Keywords:** Depression, Nutrition, Weight changes, Food intake, Physical activity, Psychiatric drugs

## Abstract

**Background &Aims:**

Depression may lead to obesity, just as obesity can contribute to the disease; yet, changes in the dietary pattern and food habits in depressive syndromes have been scantily investigated. We aimed to identify possible associations between nutritional factors and depressive disorder.

**Methods:**

This cross sectional study included 127 consecutive ambulatory adult patients with depression (DSM-IV), under psychiatric treatment. All study parameters were classified according to sex & age: BMI, waist circumference, %fat mass, food intake & physical activity.

**Results:**

Patients’ mean age was 48 ± 13 (18–81) yrs, 94% were women. Overweight/obesity was found in 72% of the cohort, 72% had excessive fat mass & 69% had a waist circumference above the maximum cut-off value. Longer disease was associated with higher BMI +%fat mass, p < 0.003. Weight gain during illness was registered in 87%; just 12% lost weight, though undernutrition did not occur. Weight gain and greater fat mass were related with higher BMI, p = 0.002. The pattern of food intake was poor, monotonous and inadequate in 59% of patients; there was also a regular consumption of hypercaloric foods by 78% pts. Overall, the usual diet was associated with weight gain, p = 0.002. Antidepressants (75%) and benzodiazepines (72%) were prevalent; these drugs were associated with weight gain, p = 0.01; 80% pts did not practice any physical activity.

**Conclusions:**

There was a positive association with overweight/obesity: a striking & clinically worrying prevalence of high fat mass, abdominal fat, weight gain, poor nutritional intake and sedentarism. This unhealthy pattern points towards the need of a multidisciplinary approach to promote healthy lifestyles that may help depressive disorder management.

## Introduction

Psychological wellbeing is essential for Quality of Life, in which an adequate nutritional status plays an important role [5]. The 4^th^ National Health Inquiry showed that 8.3% of the Portuguese population suffered from depressive disorders, with a higher prevalence in women [6]. These diseases may be classified according to severity, chronicity and symptoms’ persistence [7]. As such, the classification of minor depressive disorders, e.g. dysthymia, is used when 2 to 4 depressive symptoms are present for a minimum of 2 weeks [7, 8]. If ≥4 symptoms are present for more than 2 weeks, then we have a major depressive disorder [7, 9]. Changes in food intake habits can range from anorexia to excessive and compulsion intake, e.g. hyperphagia. Hence, there can be considerable weight gain variations during the disease; specific conditions contribute to weight gain such as: binge eating disorders, predominantly shown in major depression [2, 10], the side effects of psychotropic drugs, appetite and weight stimulation [11]. The evidence shows that obesity is associated with various co-morbidities, which have a potential influence in mortality [1]. On the other hand, a relationship was identified between obesity and psychological morbidity, suggesting that overweight/obese patients frequently show signs and/or symptoms of depressive nature [1–3], thus suggesting that depressed and obese patients have a high risk of developing cardiovascular diseases and diabetes *mellitus* [4]. The association between obesity and depression is still not well known, but suggested to be bidirectional: obesity can cause depressive alterations, and depressive disorders can lead to obesity. Understanding the factors associated to this relationship is crucial for the effective treatment of both diseases [1, 4, 12–16].

This study in patients with diagnosed depression, aimed: 1) to characterize their nutritional pattern: nutritional status, dietary habits and body composition; 2) to investigate factors that influence weight gain, e.g. food intake, physical activity, psychotropic drugs. The ultimate objective is foster the integration of nutrition and food education in the treatment of these patients, allowing on one hand the prevention of further weight gain and co-morbidities’ development, and on the other, to improve the patients´ health status and Quality of Life.

## Methods

### Study design and patient sample

This cross sectional study, approved by the National Committee for Data Protection and by the Ethics Committee of *Hospital Nossa Senhora do Rosário*, EPE *Barreiro* - Portugal, was conducted in accordance with the Helsinki Declaration, adopted by the World Medical Association in 1964, amended in 1975 and updated in 2002; all participants gave their informed consent. This study was carried out in ambulatory patients under psychiatric treatments at individualized consultations from March 2012 and December 2012. A Dietetics consultation was designed specifically for the data collection in this study. Exclusion criteria comprised patients who had been hospitalized within the previous 6 months and patients who had received drug therapy for weight reduction. Data were recorded on individual forms pre-constructed for statistical analysis.

### Study measures

Demographics (sex, age) and clinical data (disease progression, co-morbidities and psychiatric drugs) were obtained by reviewing patients’ records; anthropometric parameters, food intake and physical activity were assessed by validated questionnaires. Data collection and evaluations were always performed by the same trained dietitian (JC).

#### Nutritional status: anthropometry

Height was measured in the standing position using a stadiometer, a measurement further confirmed by comparison with patients’ Identity Card; no discrepancies were found. Weight and body composition was determined with a calibrated *Tanita Body Composition Analyser BC-418*®, with the patients shoeless and with light clothing. Body Mass Index [BMI = weight (kg)/height(m)^2^] was classified according to World Health Organization criteria [17]: undernutrition if <18.5 kg/m^2^; adequate if ≥18.5–24.9 kg/m^2^; overweight if ≥25–29.9 kg/m^2^; and obesity if ≥30 kg/m^2^. Weight changes were calculated by comparing patients’ self-reported usual weight in the previous 6 months, with the patients’ current weight. Percentage of weight loss or weight gain was also calculated. Weight gain was defined and considered significant if the patient has gained more than 4 kg since the last evaluation.

Waist circumference was determined with the patient in expiration, measured in the midpoint between the iliac crest and last floating rib, in a horizontal plane using a non-stretchable flexible tape [18]. Values were categorized according to sex, taking into account the international cut-offs which evaluate patients cardio-metabolic risk: *≥*102 cm in men and *≥*88 cm in women [19, 20]. Body fat mass was assessed by bipolar bioimpedance analysis (*Tanita Body Composition Analyzer* BC-418®). Results were expressed as %fat mass and compared with normal international cut-offs: between 21%-33% in women and 10-25% in man [21].

### Dietary habits

A structured questionnaire comprising objective questions with open answers was used, to assess food choices and to characterize the patients’ dietary patterns. In detail, patients were asked specific questions about their usual number of meals per day and the time interval between meals. Besides this information, a 24 hour recall food questionnaire was applied [34]. A score was assigned to each of the parameters studied: 1) number of meals per day being defined as adequate if ≥4meals/day and inadequate if ≤3 [23, 24]; 2) the interval between meals (maximum 4 points) being defined as adequate a maximum of 3.5 hours between meals and inadequate if the interval was greater than this cut-off [23, 24], 3) quality and quantity of foods consumed (maximum 6 points) were compared to the recommended number of portions to consume per day, validated for the Portuguese population [24]; these punctuations were chosen in accordance with the practice of recommendations for a healthy diet [22–24]. In detail, the allocation of points was performed at the end of the questionnaire, reaching a total maximum of 20 points. In summary, points were allocated for consuming a certain number of items from each food group, according the above mentioned recommendations [22–24].

After completion of the questionnaire, and according to the diet intake of the patients, points were allocated and interpreted as: higher scores were associated with appropriate dietary habits. This method was based on the recommendations given by the WHO [22–24]. Dietary habits were thus structured and valued as: adequate (15–20 points) [22–24], barely adequate (9–14 points) [22–24] or inadequate (0–8 points) [22–24]. Daily intake high caloric dense foods, defined as providing more than 300 kcal in a piece of food [4], sugary beverages and high fat foods were valued, due to the known relationship between a higher intake of these foods and depressive states. Episodes of binge eating (eating more than 4 pieces of high calorically dense foods in a row, one or more times per day) [23–25] during the day and night were assessed with open questions. Of note that patients’ medical records were reviewed, and there was never any information regarding advises on lifestyle changes. Patients were never advised to improve their lifestyle habits.

### Physical activity

Physical activity was evaluated through a structured questionnaire with points to different variables [35]. They comprised: type, weekly duration (maximum of 5 points), frequency (maximum of 5 points) and intensity of exercise – 10 points as the total maximum. The higher score was considered the most suitable physical activity. Physical activity pattern was classified as: adequate (8–10 points), barely adequate (5–7 points) and inadequate (0 to 4 points).

#### Statistical analysis

Statistical analysis was performed using SPSS 16.0 for Windows (SPSS Inc, Chicago, USA 2003®) and Microsoft Office Excel 2007®. Descriptive statistics expressed in number and percentage, were used for categorical variables (sex, co-morbidities, BMI, waist circumference, weight variation and %body fat); prevalence or frequency of categorical variables which were further evaluated by the Chi-square test. Age and disease duration were expressed as mean ± standard deviation (limits). Associations between numerical and categorical variables were explored by the non-parametric Mann–Whitney *U* test and Kruskall-Wallis test. Food habits and physical activity were classified in categories (adequate, barely adequate and inadequate) to explore associations with other variables using the same tests. Spearman´s correlations were performed between nutritional and clinical parameters, and *P* values and statistical significance was set for a p value *p* < 0.05.

## Results

### Patients

We evaluated 127 adult patients (≥18 years old) of both genders, with diagnosed depressive disorder. Mean age was 48 ± 13 (18–81) years; the majority was female: 119/127 (94%) and only 8/127 (6%) were male. During this period there were 2 eligible patients that did not wish to participate in the study; we did not have any patient that fulfilled the exclusion criteria (being hospitalised within the previous 6 months, received drug therapy for weight reduction). The disease duration ranged from 2 months to 30 years, but 46% (n = 58) of the patients had been diagnosed between 1 to 5 years prior to the data collection. Regarding the previous health status, 43% (n =55/127) of patients had one or more co-morbidities.

#### Nutritional status: anthropometry

The majority of patients (72%; n = 92/127) was overweight or obese; 72% (n =91) had excessive body fat and 69% (n =87) had a waist circumference above the maximum cut-off (Table 1). Regarding weight changes during illness, 110 patients (87%) gained weight and just 16 patients (12%) lost weight. Weight gain ranged from 0.4% to 129% in comparison to their usual weight. There was a significant correlation between weight gain and longer disease duration (p = 0.038). We also found a significant correlation between weight changes and BMI (p = 0.002), since 84 patients (76.4%) who gained weight, were overweight /obese; plus, a higher BMI and greater %body fat were correlated in both sexes (p < 0.002).

**Table 1:** **Nutritional status: anthropometry**

	n (%)
**BMI (kg/m** ^**2**^ **)**	
Underweight	1 (1%)
Adequate	34 (27%)
Overweight	34 (27%)
Obesity	58 (46%)
**% body fat mass**	
<25%♂ and <33%♀	36 (28%)
≥25%♂ and ≥33%♀	91 (72%)
**Waist circumference (cm)**	
<102♂ and <88♀	40 (31%)
≥102♂ and ≥88♀	87 (69%)
**Weight changes**	
Lost weight	16 (12%)
Maintained weight	1 (1%)
Gained weight	110 (87%)

Weight loss: was considered in the previous 6 months; it was considered significant if ≥5% of the usual weight; weight gain: was defined and considered significant if the patient has gained more than 4 kg since the last evaluation.

### Dietary habits

In this population, 59% (n = 75) of patients had a barely adequate or inadequate diet (Table 2); 67 patients (53%) had an adequate number of meals and 49 patients (46%) had an adequate break between meals, while 68 patients (54%) had a barely adequate or inadequate break between meals.

**Table 2:** **Dietary habits, physical activity and psychotropic drugs**

	n (%)	n (%)		n (%)
**Dietary habits**		**Physical activity**	**Psychotropic drugs**	**95 (75%)**
Adequate	52 (41%)	16 (12%)	Antidepressants	92 (72%)
Barely adequate	35 (28%)	10 (8%)	Anxiolytics	39 (32%)
Inadequate	40 (31%)	101 (80%)	Antipsychotics	23 (17%)
**Number of meals**			Mood stabilisers	2 (2%)
Adequate (5 – 7 meals/day)	53 (41%)		Muscle relaxants	
Barely adequate (3 – 4 meals/day)	67 (53%)			
Inadequate (<3 ou >7 meals/day)	7 (6%)			
**Interval between meals**				
Adequate (2 – 3.5 hours)	59 (47%)			
Barely adequate (4 – 5 hours)	43 (33%)			
Inadequate (<2 – >5 hours)	25 (20%)			
**High caloric density foods**				
Yes	99 (78%)			
No	28 (23%)			
**Binge eating behaviour**				
Yes	49 (39%)			
No	78 (61%)			

Adequate (15–20 points) [22–24]; barely adequate (9–14 points) [22–24] and inadequate (0–8 points) [22–24] according to the WHO criteria. High caloric density foods: >300kcal per piece of food [4]; binge eating episodes: eating ≥4 portions of 200 grams each of a high caloric density food, all eaten without intervals between each other; to be considered binge, these episodes happen daily, more than once a day or during the night [13]. Adequate (30 minutes of moderate activity 5 days/week, or 30 minutes of intense activity 2 days/week; barely adequate (half of the values presented for adequate) and inadequate (sedentarism or absence of daily physical activity) according to the WHO criteria.

Regarding the quality of food daily ingested, 55% (n = 70) ate the foods listed in the “Portuguese Food Wheel” according to the recommended number of portions per day validated for the Portuguese population, while 45% (n = 57) had a food intake barely adequate or inadequate. 46 of the 70 patients (66%) who had eaten the foods listed in the “Portuguese Food Wheel” had an adequate number of meals and break between meals (p < 0.04). On the other hand, we found that 42 of 57 (74%) patients who had a barely adequate or inadequate food intake had also a barely adequate or inadequate number of meals and break between meals (p < 0.05). Only 11 of these patients (19%) had an adequate number of meals and break between meals (p < 0.05).

Concerning the quantity of food ingested, only 7% (n = 9) respected the recommendations. These patients had also eaten all the foods recommended by the “Portuguese Food Wheel”, and had made an adequate number of meals and break between meals (p < 0.04). However, 93% (n = 118) ate barely adequate or inadequate quantities of food and 41 of them (35%) had also an inadequate number of meals and break between meals, and followed the food quality recommended by the “Food Portuguese Wheel” (p < 0.05).

There was a significant correlation between food habits and weight changes (p = 0.05), since 55% (n = 69/110) of patients who gained weight had barely adequate and inadequate food habits. On the other hand, 62% (n = 10/16) of patients who decreased weight had adequate food habits.

The intake of high density foods was predominant in most patients (n = 99, 78%), being sweets, such as chocolate, the most consumed, p < 0.05. Binge eating was absent in the majority of patients (61%). Between the consumption of high density foods and the %body fat mass in women (p = 0.013) was found a significant correlation, as well as the waist circumference (p = 0.013) and BMI (p = 0.086); however, the correlation was not significant to weight changes (p = 0.721). Additionally, we did not find a significant correlation between the intake of high density foods and disease duration (p = 0.736).

### Physical activity

The majority of patients (n = 101/127, 80%) did not exercise.; 12% (n = 16) of patients who regularly practiced physical activity, practiced 30 minutes of moderate activity 5 days/week, or 30 minutes of intense activity 2 days/week. Additionally, 88% (n = 97/110) of patients who gained weight did not practice any physical activity, and in contrast, 19% (n = 3/16) of patients who decreased their weight, had adequate physical activity habits. We explored these associations, and a concordance analysis showed that patients who gained weight did not practice physical activity (k = 0.76, p < 0.02) and patients who lost weight were physically active (k = 0.78, p < 0.01). There was no association between physical activity and BMI, %body fat mass or waist circumference (p = 0.444).

### Psychotropic drugs

The majority of patients were submitted to pharmacological treatment with psychotropic drugs, mainly antidepressants (n = 95) and benzodiazepines (n = 92). We found that 81 patients medicated with antidepressants gained weight (p = 0.002) and patients under antidepressive therapy had a higher BMI; in detail, 74 patients (79%) taking antidepressants were overweight/obese (p = 0.013). No association was found between antidepressants and increased waist circumference or higher%body fat mass. Of the 23 patients that were taking mood stabilizers, nearly all (n = 22) increased their weight (p = 0.01); 35 patients (n = 39) taking antipsychotics also gained weight (p = 0.001). Thus, a significant association between psychotropic drugs and weight changes was demonstrated.

After analyzing the cohort according to the disease duration, we separated the patients into 2 groups: the first group had been diagnosed less than 5 years ago and the second group had the disease for more 5 years. This “division” facilitated the clinical interpretation of the eating behavioural changes and co-morbidities associated with age and disease duration as well. In what concerns BMI, there was a higher the percentage of overweight/obese patients was higher in the second group (76%) that had the disease for a longer time *vs* patients in the first group (70%). Similar results were found for %body fat: the second group had higher (79%) % body fat *vs* the first group (71%). The statistical difference was negligible in what concerns to waist circumference.

## Discussion

Although conscious of the limitations of the present study, e.g. study design (cross-sectional) and clinical characteristics of the patient’ population, we found relevant that this paper showed a high percentage of patients with overweight/obesity (72%), increased cardio-metabolic risk according to their waist circumference (69%) and excessive body fat (72%). Furthermore, the majority of patients (87%) gained weight during the disease and there was a positive correlation between weight gain, higher BMI and longer disease duration. To provide an idea of the relevance of these data, preliminary data in the healthy Portuguese population show that waist circumference is almost half than the values shown in this study, weight gain does not have the magnitude shown by these patients, binge eating is not an issue and the frequency of intake of high caloric density foods is less than half (work under development).

Depression can effectively affect patients’ nutritional status, with serious consequences in the patients’ future health. Noteworthy is the fact that 31% of patients had a BMI above the maximum cut-off, excessive %body fat and waist circumference were as well above of the recommended upper limit. These patients also had gained weight during their disease, mostly due to their inadequate food habits and to sedentarism. Additionally, the prevalence of co-morbidities like high blood pressure and dyslipidaemia in this cohort cannot be disregarded, as such co-morbidities are risk factors for cardiovascular diseases [4, 14].

Inadequate or barely adequate food habits were found in 59% of patients, whilst 53% of patients followed an adequate number of meals per day; 46% made an adequate break between meals and 55% ate preferably foods as recommended by the “*Portuguese Food Wheel*” for health promotion; yet only 7% of patients had a food intake that concurred with the guidelines. Indeed, the greatest concern in these patients was their excessive food intake. In agreement with the literature, appetite and food behaviour are modified in depressive states, e.g. patients are more prone to increase the consumption of foods rich in sugar and/or chocolate [26–29, 32, 33]. In the present study, 78% of patients ate high caloric dense foods, especially those rich in sugar and chocolate. Body fat and waist circumference were significantly correlated with this excessive intake of high calorie dense foods [32].

The implementation and promotion of physical activity in the treatment of patients with depression may be beneficial [30–33]. However, we found that the majority of patients (87%) were sedentary and only 13% had any physical activities, such as the WHO recommendations of ≥30 minutes of moderate physical activity 5 days a week or 20 minutes of intense physical activity 3 days a week [1, 30–33]. Although we did not find an association with nutrition parameters, we did find a concordance between weight gain and low physical activity. The lack of significance in the association analysis may be due to the number of patients included.

In what concerns psychotropic, in this paper we showed that drug therapy was varied and monotherapy was rare. The most Drug regimens were usually combinations of antidepressants, anxiolytics, sedatives, hypnotics, antipsychotics and mood stabilizers. Antidepressants were the most prescribed drugs (75%), followed by antipsychotics in 32% of patients, and mood stabilizers in 17%. In addition to all the factors found in this study that were associated with an unhealthy nutritional status, we also found that psychotropic drugs, such as antidepressants, antipsychotics and mood stabilizers, were associated with weight gain and higher BMI.

The results of this study suggest the described association between overweight/obesity and depressive disorders. It is essential to assess patients’ overall nutritional dimensions, to identify and understand factors underlying this association, in order to implement an effective treatment of both the depressive disorder and the nutritional deficiencies [36]. On the other hand, this study also showed the patients’ perceptions that the treatment with psychiatric drugs contributed to their weight gain.

## Conclusion

This paper was useful to show that nutritional parameters, physical activity and metabolic changes were of low priority, or even completely ignored in the treatment of these patients with depression. Clinicians have to be fully aware of all side effects of pharmacological therapy, in order to decide the most adequate and effective pharmacological strategy. These patients should be advised to adopt a healthy lifestyle, with an adequate diet and regular physical activity, to prevent and treat possible overweight/obesity and metabolic co-morbidities. These patients would benefit from a holistic and multidisciplinary approach that has to include nutrition professionals trained in psychiatric disorders, to promote and optimise the benefits of physical activity and adequate eating, also in modulating their depressive disease. As clinicians know, self-reported weight may potentially influence results, since patients tend to report a lower weight than the real one, thus underestimating the significance of statistical associations and overall weight pattern of this patient’ population. We are aware that this factor may be a limitation of this study, thus usual weight was always self-reported after weight was measured. Notwithstanding, we needed to have a measure of comparison that could be easily obtained in the clinical routine where this study was developed.
